# A comparative analysis of histologic types of thyroid cancer between career firefighters and other occupational groups in Florida

**DOI:** 10.1186/s12902-022-01104-5

**Published:** 2022-09-02

**Authors:** Kemi Ogunsina, Tulay Koru-Sengul, Valentina Rodriguez, Alberto J. Caban-Martinez, Natasha Schaefer-Solle, Soyeon Ahn, Erin N. Kobetz, Monique N. Hernandez, David J. Lee

**Affiliations:** 1 Department of Public Health Sciences, Leonard M. Miller School of Medicine University of Miami, Miami, FL USA; 2 Sylvester Comprehensive Cancer Center, Leonard M. Miller School of Medicine University of Miami, Miami, FL USA; 3 Division of Endocrinology, Diabetes, and Metabolism, Department of Medicine Leonard M. Miller School of Medicine University of Miami, Miami, FL USA; 4 Department of Medicine Leonard M. Miller School of Medicine University of Miami, Miami, FL USA; 5 Department of Educational and Psychological Studies, School of Education and Human Development University of Miami, Miami, FL USA; 6 Florida Cancer Data System, Sylvester Comprehensive Cancer Center, Leonard M. Miller School of Medicine University of Miami, Miami, FL USA

**Keywords:** Thyroid cancer, Firefighters, Histologic types, Papillary, Occupation

## Abstract

**Background:**

Florida Firefighters experience a higher risk of thyroid cancer than non-firefighters. This study examines whether the histologic types and tumor stage of thyroid cancer is different among firefighters compared to other occupational groups.

**Methods:**

Eligible cases were firefighters (*n* = 120) identified in a linkage of Florida Cancer Data System (FCDS) registry records (1981–2014) and Florida State Fire Marshal’s Office employment and certification records, and non-firefighters classified into: blue-collar (*n* = 655), service (*n* = 834), white-collar (*n* = 4,893), and other (*n* = 1,789). Differences in thyroid histologic type (papillary, follicular, and rare/other less common forms of thyroid cancer), tumor stage, and age at diagnosis were evaluated using multinomial logistic regression models comparing blue-collar, service, white-collar, and other occupational groups with firefighters. Univariate odds ratios as well as odds ratios adjusted for age, gender, race, tumor stage, and year of diagnosis (aOR) and 95% confidence intervals (95%CI) were reported.

**Results:**

Service (aOR = 4.12; 95%CI: 1.25—13.65), white-collar (aOR = 3.51; 95%CI: 1.08—11.36), and blue-collar (aOR = 4.59; 95%CI: 1.40—15.07) workers had significantly higher odds of being diagnosed with rare histologic types of thyroid cancer vs papillary type compared to firefighters. Service (aOR = 0.42; 95%CI: 0.27—0.66), white-collar (aOR = 0.39; 95%CI: 0.26—0.59), blue-collar (aOR = 0.36; 95%CI: 0.23—0.56), and other (aOR = 0.34; 95%CI: 0.22—0.53) occupational groups have a significantly lower odds of being diagnosed with rare vs papillary type at a younger age (30—49 years) vs 50—69 years compared to firefighters. However, stage at diagnosis was not significantly different among occupational groups.

**Conclusion:**

Firefighters diagnosed with thyroid cancer experience a higher odds of papillary compared to rare histologic types of thyroid cancer relative to other workers; there is no evidence of an increased odds of late-stage diagnosis in firefighters relative to other worker groups. Firefighters may benefit from routine screening and active surveillance of suspected thyroid tumors especially given the excellent treatment outcomes available for those diagnosed with early-stage papillary thyroid tumors.

**Supplementary Information:**

The online version contains supplementary material available at 10.1186/s12902-022-01104-5.

## Background

Thyroid cancer is the most common endocrine cancer globally [1]. The estimated number of new thyroid cancer cases diagnosed in the United States (US) in 2021 is approximately 44,280 [2]. The incidence of thyroid cancer has tripled in the last four decades largely due to papillary thyroid cancer diagnoses (PTC) [3]. This increase in incidence is arguably due to increased diagnosis with the use of more sensitive diagnostic techniques [2, 4]. However, a true rise in thyroid cancer cases exists as increases have been reported in the advanced stage of papillary thyroid cancer and in mortality [3]. Histologic types of thyroid cancer include papillary, follicular, Hurtle, poorly differentiated, medullary, and anaplastic thyroid cancer, with papillary thyroid cancer (PTC) and follicular thyroid cancer (FTC) being the most common [5].

Florida firefighters experience a higher incidence of thyroid cancer than the general Florida population. Analysis of data from the Florida Cancer Data System (FCDS) show that, the adjusted odds of thyroid cancer for career male and female firefighters is more than two times that of non-firefighters; 2.17 (95% Confidence Interval: 1.78–2.66), and 2.42 (1.56–3.74), respectively [6]. This association is notably stronger than pooled estimates in recent meta-analysis of firefighter cancer studies (1.22; 1.01–1.48) [7], and (1.26; 0.98–1.54) [8]. The increased risk of thyroid cancer among Florida firefighters may be associated with exposure to carcinogens present in flame retardants, e.g., Poly Brominated Diphenyl Ether (PBDE), which is known to be genotoxic and cause thyroid [9] and liver tumors in animal models [10]. Studies have shown BDE 28, BDE 209 and tris(2-chloroehyl)phosphate are associated with higher odds of papillary thyroid cancer in humans [11, 12]. However, a meta-analysis by Alsen et. al and a study by Marotta et. al suggests more studies are needed to properly establish the association of endocrine disrupting chemicals (EDCs) and thyroid cancer [13, 14]. Firefighter exposure to flame retardants occurs through inhalation, ingestion, dermal contact, and contaminated personal protective gear used in the course of their work [15, 16]. While firefighters may be at increased risk of thyroid cancer due to these exposures, it is unclear if they experience a different distribution of histologic types of thyroid cancer or a difference in age or stage at diagnosis due to a combination of their individual and unique exposures to occupational carcinogens. Documentation of histologic types of thyroid cancer in this at-risk occupational group is therefore essential.

Currently, there is not enough scientific evidence to support the need for routine thyroid screening among firefighters. Thus, greater knowledge about thyroid cancer histologic types and stage at the time of cancer diagnosis can help inform policymakers decision making regarding recommendations for thyroid screening in this occupational group. In this study, we examined the histologic distribution of thyroid cancer among firefighters compared to other broad occupational groups. We hypothesize that firefighters would have a higher incidence of papillary thyroid cancer because of their exposure to flame retardants and other carcinogens and that they would be diagnosed at a younger age and an earlier cancer stage than other occupational groups. Findings from this study may also help to shed light on the high incidence of thyroid cancer among firefighters.

## Materials and methods

### Study population

**Fig. 1:**
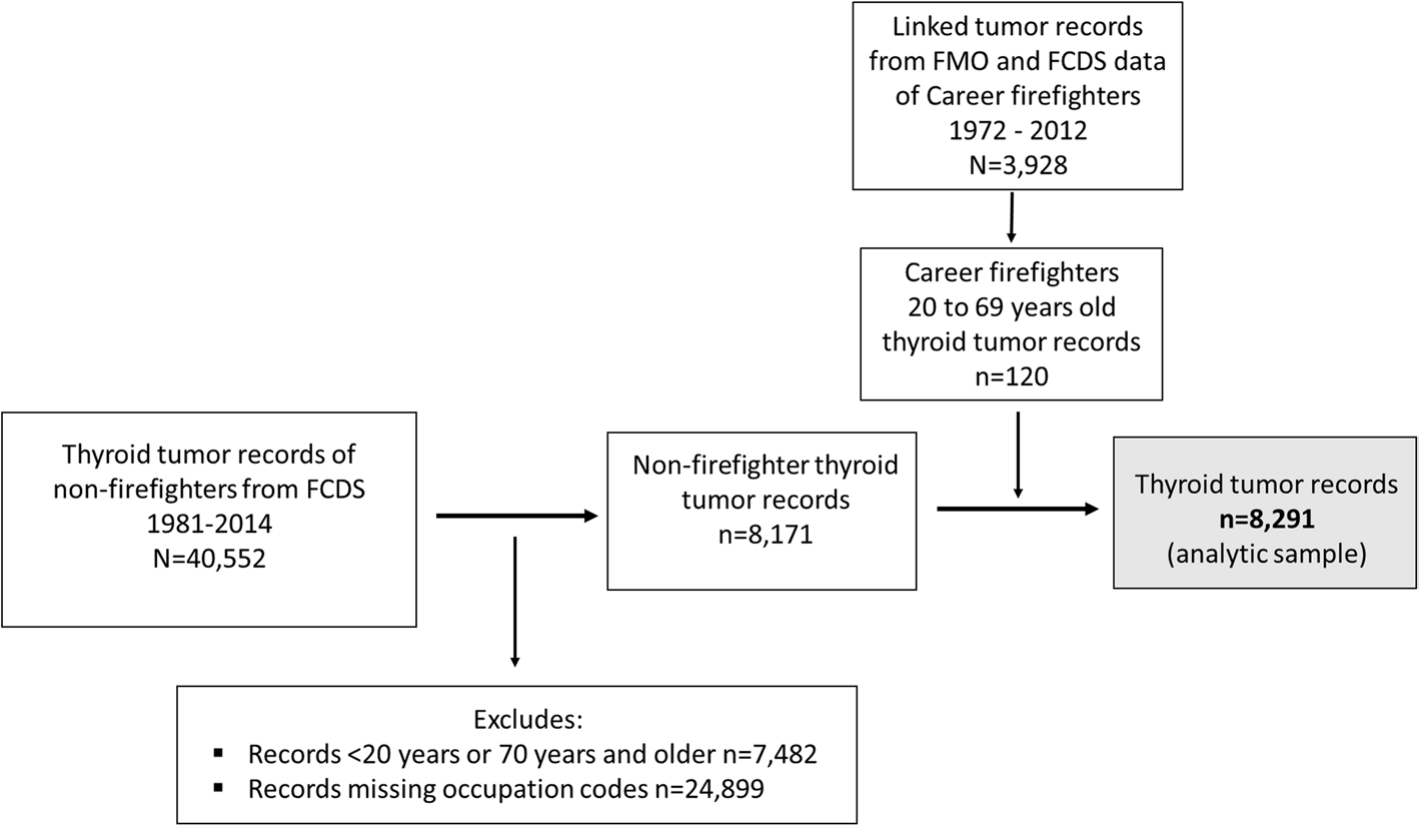
A flow chart showing the derivation of career firefighters and non-firefighter thyroid tumor records from the Fire Marshalls Office (FMO) and Florida Cancer Data System (FCDS), and the thyroid tumor analytic sample used in this study

The study population consisted of incidence thyroid tumor records from the Florida Cancer Data System (FCDS) registry from 1981 – 2014. Since 1981, the FCDS has served as the legislatively mandated, population-based cancer registry for Florida collecting all newly diagnosed cancer cases in the state. It operates under the auspices of the Florida Department of Health as part of the National Program of Cancer Registries (NPCR) [17]. Florida State Fire Marshal’s Office (FMO) employment and certification records of career firefighters (1972–2012) were linked with FCDS tumor registry records (*n* = 3,928); detailed information regarding the linkage is published elsewhere [18]. The FMO-FCDS linked data is only available up to the year 2014. A total of 40,672 tumor records with a thyroid cancer diagnosis were identified using the primary site code C73.9 for thyroid in combination with the International Classification of Diseases for Oncology, third edition (ICD-O-3) used principally in cancer registries for coding the histology of neoplasms. Exclusion criteria include 1) benign/stage 0 tumors; 2) tumor records of individuals younger than 18 years or 70 years and older (*n* = 7,482); and 3) tumor records missing census 2010 occupation codes (*n* = 24,899). A total of 8,291 participants with thyroid cancer as their primary cancer were retained for analyses following the application of an exclusionary criteria. A study flow chart depicting the linkage and data used for this analysis is provided in Fig. 1. A comparison of sociodemographic and clinical characteristics of excluded tumor records compared to our analytic sample can be found in Supplemental Table 1.

### Study measures

#### Histologic types

Diagnoses of all cancer sites in the FCDS registry are based on histopathological examination of affected tissues. Histopathologic diagnoses are coded according to the ICD-O-3 [19]. Only tumors with histologic codes and behavior type 3 (malignant) were included in the study data. For this analysis, we categorized histologic types into 3 main categories namely, papillary thyroid carcinoma (ICD-O-3 histology codes 8050, 8260 – 8263, 8340 – 8344, and 8504), follicular thyroid carcinoma (ICD-O-3 codes 8330 – 8332, and 8335), and others (ICD-O-3 codes 8010, 8012, 8020 – 8022, 8030 – 8033, 8035, 8041, 8046, 8070 – 8072, 8074, 8076, 8130, 8140, 8190, 8230, 8240, 8246, 8255, 8290, 8310, 8337, 8345 – 8347, 8350, 8430, 8450, 8460, 8480, 8481, 8510, 8550, 8589, 8800, 8802, 8810, 8830, 8890, 8901, 8940, 8980, 9120, and 9150). The category ‘rare’ includes poorly differentiated (medullary carcinoma, anaplastic carcinoma,), poorly differentiated (oxyphilic adenocarcinoma) other rare forms, and unknown types of thyroid cancers. These are the aggressive types of thyroid cancer. Mixed follicular-papillary carcinoma and follicular variant of papillary carcinoma were categorized as papillary carcinomas.

#### Cancer staging

The FCDS defined cancer staging using the Surveillance, Epidemiology, and End Results Program (SEER) summary stage 2000 [20]. The summary staging is a basic way of categorizing how far a cancer has spread from its point of origin using all information available in the medical record. We condensed SEER stages 0 and 1 into an early stage for malignancies confined to the thyroid or invading the capsule but not beyond, and SEER stage 2 to 7 as late-stage for tumors with regional and distant metastasis. Benign tumors (stage 8) were excluded from registry data as stated previously, and stage 9 as unknown stage for tumor records with inadequate information for staging were also excluded.

#### Occupational groups

Firefighters were identified via the linkage with Florida firefighter employment and certification records as described above. Using the 2010 census occupation codes for all other non-firefighter thyroid cancer cases, occupation is classified into one of 472 broad occupations according to their occupational definition. Based on a cross-walk of the 2010 census occupation codes and the conventional U.S classification of occupation, we defined four major occupational categories of “service”, “white-collar”, “blue-collar”, and “farmworkers” [21, 22]. Because “farmworkers” represented a small group of cases, we included them with homemakers, students, retired, and disabled in the category “other”. The white-collar group included occupations such as executive professional, managerial, administrative, technical, clerical, and sales positions. The blue-collar group included occupations in precision production, construction, machine operators, assemblers and inspectors, transportation, material moving occupations, craft and repair, helpers, and laborers. The service group included occupations in protective occupations such as the police, and non-protective occupations such as the service industry, medical field, and private household occupations.

#### Other covariates

Other variables included in this analysis are race categorized into White, non-White which includes individuals with multiple races, American Indian or Alaska Native, Black, Asian, Native Hawaiian or other Pacific Islander, and unknown race which included individuals missing race information. Ethnicity was categorized into Hispanic, non-Hispanic, and unknown which included records missing ethnicity information. Both race and ethnicity were self-reported data obtained from the registry. Cancer diagnosis year was categorized into three groups based on 10-year periods i.e., 1981–1991, 1992–2002, and 2003–2014.

### Statistical analysis

The distribution of sociodemographic and tumor characteristics comparing firefighters and other occupational groups was analyzed. The differences in the mean age at cancer diagnosis for firefighters compared to non-firefighter occupational groups were examined using an ANOVA test for mean, while differences in proportions from categorical variables were examined with Chi-Squared tests. Furthermore, analyses were conducted to examine differences in the overall incidence of histologic types, and distribution of histologic types of thyroid cancer stratified by age, gender, and tumor stage among firefighters compared to other non-firefighting occupational groups. This was also analyzed using a Chi-Squared test. Subsequently, multiple pairwise comparisons were employed using t-test for mean age and Fisher’s Exact test for categorical variables to compare differences in proportions for firefighters with each non-firefighting occupational group with post-hoc Bonferroni correction. Sample sizes less than 10 were not reported due to FCDS policy on confidentiality rules. We reported frequencies, percentages, and *p*-values from both Chi-squared, and Bonferroni adjustment.

Outcome variables were multinomial categorical variables namely: histologic type (papillary, follicular, and rare), age (18 – 29, 30 – 49, and 50 – 69), and tumor stage (early, late, and unknown stage) of thyroid cancer at diagnosis. Therefore, we employed a series of multinomial logistic regression modeling to calculate adjusted odds ratio (aOR) and 95% confidence intervals (95% CI) for our 3 outcome variables. In our first model, we use occupation as a main predictor, assessing the odds of follicular vs papillary (reference group), and rare types of thyroid cancer vs papillary simultaneously among each of the occupational groups compared to firefighters (reference group). Variables adjusted for were selected based on statistical significance and literature reviews [2, 23]. We applied three models: An unadjusted model, a full model adjusting for age, gender, race, ethnicity, tumor stage and year of diagnosis, and a final model adjusting for all other variables in the full model except for ethnicity. Additional adjustment for ethnicity did not change the results, and the variable was not significant considering the Wald test statistics; therefore, this variable was not included in the final model.

We repeated the same multinomial regression model steps as above for our second model with the outcome age group at cancer diagnosis and used age group 50 – 69 years as our reference group. For our third multinomial model with tumor stage as our outcome, we used late-stage as our reference group comparing firefighters with other occupational groups. Further, we conducted additional analysis using binomial logistic regression to examine the odds of differentiated (papillary and follicular) compared to rare type thyroid cancer. For the binary regression we also examined the odds of diagnosis occurring at age 18 – 49 years compared to 50 – 69 years, and then examined the odds of stage at diagnosis being other (early or unknown) compared to late-stage, using firefighters as our reference group compared to other occupation groups (Supplemental Table 2).

Due to the large proportion of male firefighters (79.2%) compared to other occupational groups (20.2% service, 20.7% white-collar, 63.8% blue-collar and 8.4% others), and the large proportion of Whites among firefighters compared to other more heterogenous occupational groups, we conducted a sensitivity analysis using a multinomial model to examine the prevalence of thyroid histological types only among white males across occupational groups (Supplemental Table 3).

All tests were two-sided with type-1 error rate of 5%. Because of multiple comparisons with other occupational groups, we applied Bonferroni-adjusted type-1 error of 0.05/24 = 0.0021, and type-1 error of 0.05/36 = 0.00139 to the multiple pairwise comparisons using Fisher’s Exact test in Tables 1 and 2 respectively. This study was approved by the Florida Department of Health and University of Miami Institutional Review Board (IRB). All statistical analysis was conducted using SAS version 9.4 (SAS Institute Inc. Cary, NC).

**Table 1:** Sociodemographic and tumor characteristics of firefighters and other occupation groups in the FCDS 1981–2014; Thyroid tumor analytic dataset

**Characteristics**		**Occupation**				
	**Total** *n* = 8,291	**Firefighters** *n* = 120 (1.5%)	**Service** *n* = 834, (10.1%)	**White-collar** *n* = 4,893, (59.0%)	**Blue-collar** *n* = 655, (7.9%)	**Other **^**a**^ *n* = 1,789 (21.6%)
**Mean age (SD) **^**b**^	46.8 (12.3)	43.8 (10.7)	46.3 (12.1)	47.3 (11.8)	50.2 (11.4)	44.7 (14.0)
***p*-value**	** < 0.001**^e^	ref	0.047	**0.001**	** < 0.001**	0.487
**Age categories n (%)**						
***p*-value**	** < 0.001** ^e^	ref	** < 0.001**	** < 0.001**	** < 0.001**	** < 0.001**
18 to 29	828 (10.0)	13 (10.8)	90 (10.8)	378 (7.7)	31 (4.7)	316 (17.7)
30 to 49	3,804 (45.9)	72 (60.0)	397 (47.6)	2,319 (47.4)	271 (41.4)	745 (41.6)
50 to 69	3,659 (44.1)	35 (29.2)	347 (41.6)	2,196 (44.9)	353 (53.9)	728 (40.7)
**Gender n (%)**						
***p*-value**	** < 0.001** ^e^	ref	** < 0.001**	** < 0.001**	**0.001**	** < 0.001**
Male	1,845 (22.3)	95 (79.2)	167 (20.0)	1,015 (20.7)	418 (63.8)	150 (8.4)
Female	6,446 (77.7)	25 (20.8)	667 (80.0)	3,878 (79.3)	237 (36.2)	1,639 (91.6)
**Race n (%)**						
***p*-value**	** < 0.001** ^e^	ref	** < 0.001**	0.007	0.003	0.004
White	7,305 (88.1)	113 (94.2)	684 (82.0)	4,363 (89.2)	571 (87.3)	1,574 (88.0)
Non-White	938 (11.3)	– –	148 (–)	501 (10.2)	81 (–)	202 (11.3)
Unknown	48 (0.6)	– –	– –	29 (0.6)	– –	13 (0.7)
**Ethnicity n (%)**						
***P*-value**	** < 0.001** ^e^	ref	** < 0.001**	0.008	0.003	** < 0.001**
Hispanic	1,543 (18.6)	14 (11.7)	184 (22.1)	762 (15.6)	125 (19.1)	458 (25.6)
Non-Hispanic	6,696 (80.8)	104 (–)	646 (–)	4,101 (83.8)	526 (–)	1,319 (73.7)
Unknown	52 (0.6)	– –	– –	30 (0.6)	– –	12 (0.7)
**Diagnosis year n (%)**						
***P*-value**	** < 0.001** ^e^	ref	** < 0.001**	** < 0.001**	0.007	** < 0.001**
1981- 1991	222 (2.7)	– –	10 (1.2)	127 (2.6)	30 (4.6)	46 (2.6)
1992 – 2002	1,308 (15.8)	14 (–)	117 (14.0)	665 (13.6)	91 (13.9)	421 (23.5)
2003 – 2014	6,761 (81.6)	97 (80.8)	707 (84.8)	4,101 (83.8)	534 (81.5)	1,322 (74.0)
**Tumor stage n (%) **^**c**^						
***P*-value**	**0.002** ^e^	ref	**0.001**	** < 0.001**	**0.001**	**0.001**
Early	5,590 (67.4)	77 (64.2)	576 (69.1)	3,349 (68.4)	400 (61.1)	1,188 (66.4)
Late	2,331 (28.1)	42 (–)	222 (26.6)	1,343 (27.5)	217 (33.1)	507 (28.3)
Unknown	370 (4.5)	– –	36 (4.3)	201 (4.1)	38 (5.8)	94 (5.3)

**Bold** designates significant *p*-values adjusted for any Bonferroni correction

*Note:* – – Represents sample sizes less than 10, which are not reported due to FCDS confidentiality rules, – some data is suppressed to prevent back calculation of counts

Percentages may not add up to 100% due to rounding

^a^ Other occupation includes farmworkers, retired, students, housewife/homemakers, and disabled

^b^ Age is age at cancer diagnosis; SD is standard deviation

^c^ Based on SEER stage 2000, excludes stage 0, late stage includes regional and distant metastasis

^e^ Statistical comparison between all occupation groups, *p*-values are calculated either with Student’s t-test for continuous variables or with chi-squared test for independence and considered statistically significant when *p*-value < 0.05. Other *P*-values are adjusted for multiple pairwise comparison between firefighters and each occupation group using Fisher’s exact test with adjusted Bonferroni protected probability of *p* =  < 0.0021

**Table 2:** Distribution of histologic types of thyroid cancer stratified by age, gender, and tumor stage comparing firefighters with other occupation groups in the FCDS 1981–2014; Thyroid tumor analytic dataset

	**Total** *n* = 8,291	**Firefighters** *n* = 120 (1.5%)	**Service** *n* = 834 (10.1%)	**White-collar** *n* = 4,893 (59.0%)	**Blue-collar** *n* = 655 (7.9%)	**Other **^**a**^ *n* = 1,789(21.6%)
**Overall n (%)**						
Papillary	7,099 (85.6)	107 (89.2)	712 (85.4)	4,195 (85.7)	514 (78.5)	1,571 (87.8)
Follicular	476 (5.7)	– –	44 (5.3)	291 (6.0)	55 (8.5)	76 (4.3)
Rare type ^**b**^	716 (8.6)	– –	78 (9.4)	407 (8.3)	86 (13.1)	142 (7.9)
*P*-value*		ref	** < 0.001**	** < 0.001**	** < 0.001**	** < 0.001**
**Age n (%)**						
18 – 29 years old						
Papillary	735 (88.8)	13 (100.0)	81 (90.0)	337 (89.2)	28 (90.3)	276 (87.3)
Follicular	50 (6.0)	– –	– –	25 (6.6)	– –	19 (6.0)
Rare type ^**b**^	43 (5.2)	– –	– –	16 (4.2)	– –	21 (6.7)
*P*-value*		ref	0.281	0.231	0.339	0.179
30 – 49 years old						
Papillary	3,361 (88.4)	64 (88.9)	344 (86.7)	2,049 (88.4)	229 (84.5)	675 (90.6)
Follicular	202 (5.3)	– –	22 (5.4)	133 (5.7)	17 (6.3)	24 (3.2)
Rare type ^**b**^	241 (6.3)	– –	31 (7.8)	137 (5.9)	25 (9.2)	46 (6.2)
*P*-value*		ref	0.009	0.016	0.007	0.004
50 – 69 years old						
Papillary	3.003 (82.1)	30 (85.7)	287 (82.7)	1,809 (82.4)	257 (72.8)	620 (85.2)
Follicular	224 (6.1)	– –	17 (4.9)	133 (6.1)	37 (10.5)	33 (4.5)
Rare type ^**b**^	432 (11.8)	– –	43 (12.4)	254 (11.6)	59 (16.7)	75 (10.3)
*P*-value*		ref	0.006	0.008	0.003	0.007
**Gender n (%)**						
Male						
Papillary	1,483 (80.4)	84 (88.4)	138 (82.6)	817 (80.5)	320 (76.6)	124 (82.7)
Follicular	141 (7.6)	– –	– –	81 (8.0)	39 (9.3)	– –
Rare type ^**b**^	221 (12.0)	– –	24 (–)	117 (11.5)	59 (14.1)	18 (–)
*P*-value*		ref	** < 0.001**	**0.001**	** < 0.001**	** < 0.001**
Female						
Papillary	5,616 (87.1)	23 (92.0)	574 (86.1)	3,378 (87.1)	194 (81.9)	1,447 (88.3)
Follicular	335 (5.2)	– –	39 (5.9)	210 (5.4)	16 (6.8)	68 (4.2)
Rare type ^**b**^	495 (7.7)	– –	54 (8.1)	290 (7.5)	27 (11.4)	124 (7.6)
*P*-value*		ref	0.035	0.037	0.017	0.031
**Tumor stage n (%)**						
Early stage						
Papillary	4,899 (87.6)	68 (88.3)	503 (87.3)	2,927 (87.4)	330 (82.5)	1,071 (90.2)
Follicular	385 (6.9)	– –	38 (6.6)	239 (7.1)	39 (9.8)	61 (5.1)
Rare type ^**b**^	306 (5.5)	– –	35 (6.1)	183 (5.5)	31 (7.8)	56 (4.7)
*P*-value*		ref	0.005	0.006	0.003	0.004
Late stage						
Papillary	2,029 (87.0)	38 (90.0)	187 (84.2)	1,179 (87.8)	171 (78.8)	454 (89.6)
Follicular	64 (2.8)	– –	– –	33 (2.5)	14 (6.5)	10 (2.0)
Rare type ^**b**^	238 (10.2)	– –	30 (–)	131 (9.8)	32 (14.8)	43 (8.5)
*P*-value*		ref	0.015	0.030	0.011	0.035
Unknown stage						
Papillary	171 (46.2)	– –	22 (61.1)	89 (44.3)	13 (34.2)	46 (48.9)
Follicular	27 (7.3)	– –	– –	19 (9.5)	– –	– –
Rare type ^**b**^	172 (46.5)	– –	– –	93 (46.3)	– –	43 (–)
*P*-value*		ref	0.622	0.446	0.359	0.495

*Note:* – – Represents sample sizes less than 10, which are not reported due to FCDS confidentiality rules– some data is suppressed to prevent back calculation of counts

Percentages may not add up to 100% due to rounding

^**a**^ Other occupation includes farmworkers, retired, students, housewife/homemakers, and disabled

^**b**^ Rare types include other less common/aggressive histologic types of thyroid cancer which includes oxyphilic (27%), medullary (21%), carcinoma NOS (20%), anaplastic (10%), other rare, and unknown (22%)

^*^ *P*-value compares firefighter with each occupation group and adjusted for multiple comparison. **Bold** is significant using an adjusted Bonferroni protected probability of *p*-value =  < 0.00139

## Results

Of the 8,291 primary thyroid cancer records analyzed, 1.5% (*n* = 120) were career firefighters, 10.1% (*n* = 834) service, 59.0% (*n* = 4,893) white-collar, 7.9% (*n* = 655) blue-collar, and 21.6% (*n* = 1,789) “other” occupations. The mean (± standard deviation) age at cancer diagnosis was significantly lower for firefighters 43.8 ± 10.7 years compared to white-collar (47.3 ± 11.8 years, *p* = 0.001), and blue-collar (50.2 ± 11.4 years, *p* < 0.001) workers. Overall, tumor stage distribution (early, late, unknown) was also significantly different comparing firefighters (early stage 64.2%, late and unknown stage 35.8%) to service (69.1%, 26.6%, 4.3%; *p* = 0.001), white-collar (68.4%, 27.5%, 4.1%; *p* =  < 0.001), blue-collar (61.1%, 33.1%, 5.8%; *p* = 0.001), and other (66.4%, 28.3%, 5.3% *p* = 0.001) occupation (Table 1).

Papillary thyroid cancer was the most common histologic type for the entire study sample (85.6%) and for firefighters (89.2%). The histologic distribution of thyroid cancer (papillary, follicular, and rare) types was significantly different in firefighters (89.2%,10.8% for follicular and rare types) compared to service (85.4%, 5.3%, 9.4%; *p* =  < 0.001), white-collar (85.7%, 6.0%, 8.3%; *p* =  < 0.001), blue-collar (78.5%, 8.5%, 13.1%; *p* =  < 0.001), and other occupation (87.8%, 4.3%, 7.9%; *p* =  < 0.001). The distribution of histologic types of thyroid cancer among age groups, and tumor stage comparing firefighters with each occupation group was not statistically significant (Table 2).

Table 3 presents the adjusted odds of histologic types, age group, and tumor stage at diagnosis comparing firefighters with service, white-collar, blue-collar, and other occupation. A significantly higher odds of having a rare compared to papillary type of thyroid cancer was seen in service (aOR = 4.12; 95%CI: 1.25, 13.65), white-collar (3.51; 1.08, 11.36), and blue-collar (4.59; 1.40, 15.07) occupational groups relative to firefighters, except for other occupation, in which the odd approached significance (aOR = 3.23; 95%CI: 0.99, 10.61).

**Table 3:** Adjusted multinomial logistic regression showing the odds of histologic types, age, and stage at diagnosis of thyroid cancer in the FCDS 1981–2014; Thyroid tumor analytic dataset

Occupation groups ^a^	**Histologic sub-types**^**1**^					
	**Follicular vs Papillary**			**Rare types **^**b**^** vs Papillary**		
	**aOR**	**95% CI**	***p*-value**	**aOR**	**95% CI**	***p*-value**
Firefighters	1.00	-	-	1.00	-	-
Service	0.78	0.37, 1.63	0.505	**4.12**	**1.25, 13.65**	**0.020**
White-collar	0.90	0.47, 1.83	0.773	**3.51**	**1.08, 11.36**	**0.036**
Blue-collar	1.15	0.46, 1.80	0.702	**4.59**	**1.40, 15.07**	**0.012**
Other	0.60	0.29, 1.24	0.169	3.23	0.99, 10.61	0.053
	**Age group (years) at diagnosis**^**2**^					
	**18 – 29 vs 50—69**			**30 – 49 vs 50—69**		
Firefighters	1.00	-	-	1.00	-	-
Service	0.51	0.25, 1.02	0.058	**0.42**	**0.27, 0.66**	**0.001**
White-collar	**0.33**	**0.17, 0.65**	**0.001**	**0.39**	**0.26, 0.59**	** < 0.001**
Blue-collar	**0.22**	**0.11, 0.47**	** < 0.001**	**0.36**	**0.23, 0.56**	** < 0.001**
Other	0.74	0.37, 1.45	0.378	**0.34**	**0.22, 0.53**	** < 0.001**
	**Tumor stage **^**3**^					
	**Early vs Late**			**Unknown vs Late**		
Firefighters	1.00	-	-	1.00	-	-
Service	0.96	0.63, 1.46	0.843	5.78	0.74, 45.15	0.094
White-collar	0.90	0.61, 1.34	0.602	4.79	0.63, 36.26	0.129
Blue-collar	0.87	0.57, 1.33	0.523	4.85	0.63, 37.57	0.131
Other	0.83	0.55, 1.25	0.364	5.38	0.70, 41.19	0.106

*aOR* adjusted odds ratio, 95%CI: 95% confidence interval adjusted for ^1^ age, gender, race, tumor stage, and diagnosis year. ^2^ histologic type, gender, race, tumor stage, and diagnosis year ^3^ histologic type, age, gender, race, and diagnosis year

Significant *p*-value < 0.05

^**a**^ Other occupation includes retired, students, housewife/homemakers, farmers, and disabled

^**b**^ Rare histologic types include other less common/aggressive histologic types of thyroid cancer which includes oxyphilic (27%), medullary (21%), carcinoma NOS (20%), anaplastic (10%), other rare, and unknown (22%)

The odds of having follicular compared to papillary type of thyroid cancer, among service (0.78; 0.37, 1.63), white-collar (0.90; 0.47, 1.83), blue-collar (1.15; 0.46, 1.80), and other occupational groups (0.60; 0.29, 1.24) relative to firefighters were not statistically significantly different. Also, in Supplemental Table 2, we confirmed that the odds for differentiated (papillary and follicular combined) thyroid cancer was lower among all other occupational groups compared to firefighters.

Firefighters tend to be diagnosed at a younger age relative to other occupational groups, whereas white-collar and blue-collar workers are diagnosed at a significantly older age (see Table 1). But after adjusting for other covariates, a significantly lower odds of being diagnosed with thyroid cancer at age group18—29 years old compared to 50—69 years is seen in white-collar (aOR = 0.33; 95%CI: 0.17, 0.65), and blue-collar (0.22; 0.11, 0.47) workers relative to firefighters. Also, a significantly lower odds of being diagnosed at age group 30—49 vs. 50—69 years was seen among all occupational groups i.e., service (0.42; 0.27, 0.66), white-collar (0.39; 0.26, 0.59), blue-collar (0.36; 0.23, 0.56), and other (0.34; 0.22, 0.53) relative to firefighters. Additional analysis supports the finding that firefighters are more likely to be diagnosed at a younger age (Supplemental Table 2). After adjusting for other covariates, the odds of being diagnosed at an early or unknown stage compared to late stage was not statistically significant comparing firefighters with other occupational groups (Table 3).

## Discussion

In this study, we found a high incidence of papillary thyroid cancer overall (85.6%) and specifically among firefighters (89.2%). The mean age at diagnosis of thyroid cancer among career firefighters (44 years) is younger compared to non-firefighters overall (47 years) and particularly when compared to the mean age at diagnosis of about 47 years for white-collar, and 50 years for blue-collar occupational groups. Firefighters had a significantly higher odds of being diagnosed at a younger age group 30—49 years compared to 50—69 years relative to all other occupational groups, and at age groups, 18—29 years compared to 50—69 years relative to white-collar and blue-collar occupational groups.

The distribution of histologic types of thyroid cancer was different comparing firefighters with other groups; all occupational groups compared to firefighters had significantly higher odds of rare types of thyroid cancer vs papillary except for other occupations which were not significant. We found lower odds of follicular compared to papillary thyroid cancer among service, white-collar, and other occupations compared to firefighters; however, a higher odds was found compared to blue-collar workers, but these findings were not significant. Analysis in Supplemental Table 2 showed significantly lower odds of having differentiated (papillary, and follicular) thyroid cancer among all other occupational groups compared to firefighters. Papillary and follicular thyroid cancers are usually associated with an innocuous clinical course, though a small proportion present with a more aggressive course [24–26]. This is in contrast to medullary and anaplastic histologic types, which tend to be more lethal [24, 27].

Papillary thyroid cancer is the most prevalent histologic type of thyroid cancer overall and within each occupational group. These findings are consistent with literature that shows that papillary type thyroid cancer is the most common histologic type contributing to over 70% of thyroid cancer cases [24]. The overall five-year relative survival rate of thyroid cancer is 98% [2]; this is because most are low-risk, indolent papillary thyroid cancers, two-thirds of which are early-stage diagnoses. Treatment is usually successful for these tumors however, among people diagnosed with more advanced stage disease, five-year relative survival drops to 55% [2]. Papillary thyroid cancer tends to produce lymph node metastases and malignant central nodal metastases occur with high frequency in papillary thyroid cancer but not in the follicular variant of papillary thyroid cancer [28]. The five-year relative survival of papillary thyroid cancer overall is 88.2% and 10-year survival is 76.6% while 15-year survival drops to 35.8% [29]. The prevalence of follicular thyroid cancer is 13% [5] with a 10-year relative survival of 85% and is more aggressive than papillary type mainly because it can metastasize via vascular invasion. It often presents with metastasis at the time of diagnosis [30, 31]. The prevalence of Hurthle type is 2% with a 10-year relative survival of 76%, and the prevalence of medullary type is 4% and anaplastic 2%; both types have even lower 10-year survival rates, 75% for medullary and anaplastic being as low as 14% [5]. Although papillary thyroid carcinoma carries the most favorable prognosis among all types of thyroid cancer there are different variants with potential for aggressiveness. For example, among patients who develop metastatic papillary thyroid cancer (MPTC), the risk of death increased by nearly fivefold in patients with papillary thyroid cancer variants other than the classic papillary subtype [29].

In the general US population, the majority of the increase in thyroid cancer is observed in the papillary sub-type [32]. Though increased medical surveillance is likely contributing to this increased incidence, genetic factors, ionizing radiation exposure, and other environmental pollutants are also suspected risk factors for papillary thyroid cancer [32–34]. Firefighters have an increased odds of occupational exposure to PCBs [35], PBDEs [15, 16], and other flame retardants, which have been found to have a significant association with thyroid cancer [11, 13]. A group of firefighters in California was found to have increased serum levels of PBDEs compared to their non-firefighter counterparts [16], and another study by Hoffman et al. have also shown that individuals with higher median blood levels of PBDE were twice as likely to have papillary thyroid cancer compared to those with low blood levels of PBDE [12]. PBDEs mimic thyroid hormones (T3 and T4) by competitively binding to thyroid hormone receptors and through a series of processes that inhibit negative feedback to the thyroid gland. Their proposed mechanism of action in thyroid dysfunction is that they cause continuous stimulation of the thyroid gland and release of Thyroid Stimulating Hormone (TSH), thereby disrupting hormone homeostasis [36]. This leads to increased TSH levels and studies have shown that increased TSH levels are associated with increased odds of differentiated thyroid cancers, and advanced stage thyroid cancers [37].

Among firefighters in our study, the mean age at diagnosis is 44 years. This is younger than the mean age for the non-firefighters (47 years) and compared to each of the non-firefighting occupational groups. Firefighters have a higher odds of being diagnosed at 18—29 years compared to 50—69 years relative to white-collar and blue-collar workers, and a higher odds of being diagnosed at 30—49 years compared to 50—69 years relative to all other occupational groups. This confirms our hypothesis that firefighters would be diagnosed at a younger age. The young mean age at diagnosis of thyroid cancer among firefighters could also be associated with baseline and increased routine medical surveillance of this occupational group. Increased cancer risk awareness levels in the fire service may lead to higher cancer screening activity and subsequent higher rates of detection in younger age groups, although definitive evidence to support this hypothesis is lacking. Another reason could be increased exposure of firefighters to chemical carcinogens and radiation than non–firefighters or could be that firefighters are generally a younger population than other groups. Studies have shown an increase in the incidence of thyroid cancer among adolescents and younger people in the general population [38], and the diagnosis of thyroid cancer at younger age groups is associated with an increased incidence of a papillary type of thyroid cancer [38].

Our findings show that firefighters have a higher odds of papillary and a non-significant higher odds of follicular (both differentiated type thyroid cancer) compared to rare types of thyroid cancer and are diagnosed at a younger age. This finding is important because younger patients have an 11-fold increase in mortality between thyroid tumor stages I and II whereas there is no significant increase in mortality between stages I and II for older patients [39]. Also, at an age younger than 55 years, men have a higher odds of dying from stage I and II thyroid cancer than women [40]; This is also important because over 90% of U.S. firefighters are men [41]. In our study, we had a higher proportion of White male firefighters compared to other occupational subgroups. Hence, we examined thyroid histologic types among White men across all occupational groups (Supplemental Table 3). Findings were similar, we found lower odds of follicular and higher odds of rare types compared to papillary among each of the other occupational groups relative to firefighters, but most of the findings were not statistically significant. We also found mostly significant diagnoses in younger age groups among firefighters than in white-collar, blue-collar, and other occupational groups.

As indicated above early age at diagnosis in firefighters could reflect increased medical surveillance or access to health care among firefighters. The National Fire Protection Association (NFPA) 1582 standard recommends neck palpation for thyroid nodules as part of an annual physical examination [42]. However, fire department alignment with the NFPA 1582 standard is voluntary and percent compliance is unknown, although it is more likely in larger as opposed to smaller departments. Survey respondents at a recent Florida Fire Chiefs' Association Health and Safety conference indicated that 44% of represented fire departments engaged in cancer screening activity in the 12 months before survey administration [43]. Thus, for early diagnosis to be a result of increased medical surveillance, early detection due to increased screening would also be associated with differences in stage at diagnosis of tumors among firefighters relative to other occupational groups. A study by Lim et. al shows that enhanced health care access is associated with both younger age at diagnosis and a lower odds of presenting in a late stage of differentiated thyroid cancer [44]. However, contrary to our hypothesis, after adjusting for other covariates, we did not find significant differences in tumor stage at diagnosis of firefighters compared to other occupational groups.

The United States Preventive Services Task Force (USPSTF) recommends against screening for thyroid cancer among asymptomatic individuals (D recommendation) [45]. However, because firefighters are a high-risk population for cancers there is more attention given to regular screening. The use of third-party cancer screening services promoting different types of blood tests, breath tests, and imaging tests for cancers are also increasingly being marketed to firefighters and fire departments, although presently the International Association of Firefighters does not recommend their use in individuals without any symptoms or signs of cancer [46]. In this study, we found papillary relative to the rare types of thyroid cancers are high among firefighters, a male-dominated occupation with high exposures to EDCs, and more likely to be diagnosed in younger age groups. Studies have shown papillary thyroid cancer may metastasize to the lymph nodes with reduced survival, and the increased risk of mortality is higher in younger age groups and among men in stages I and II. Therefore, it may be beneficial for the USPSTF to consider firefighters as a high-risk group for thyroid cancer screening. Although routine screening may result in overdiagnosis and treatment, these outcomes could be minimized through baseline screening and follow-up every 5 years depending on screening results and endocrinologist recommendation.

### Strengths and limitations

This study is the first to evaluate the odds of differentiated and other rare and poorly differentiated/ aggressive histologic types of thyroid cancer comparing firefighters with non-firefighters in other occupational groups using population-based cancer registry data. This study included all certified career firefighters with a diagnosis of thyroid cancer in the state of Florida from 1981 to 2014, employing the latest linkage software (FastLink) to link records from the Florida State Fire Marshal’s Office with the FCDS data [6]. Extensive efforts are made by the Florida Cancer Registry to ensure data on all cancer patients are of the highest quality and that case ascertainment is as complete as possible. The strength of this study also includes the use of 34 years of data (1981 to 2014) for this analysis.

This study also sheds light on the proportion of missing occupation data in the Florida cancer registry. Missing occupational data is one of the challenges of examining cancer burden across occupational groups resulting in a dearth of publications in this field.

The American Joint Committee on Cancer’s (AJCC) tumor-node-metastasis (TNM) 2018 staging protocol provides clinical utility [47], and incorporates age as a determinant of the stage for differentiated thyroid cancers. This being an epidemiologic study, and due to the unavailability of AJCC/TNM stage information for the entire study period, we employed SEER summary staging condensed into three categories: i.e. early, late, and unknown stage. The main difference that could arise from the use of the SEER vs. AJCC staging is that individuals 55 years and older with tumors greater than 4 cm in size within each occupation group in our study would be included in stage I compared to stage II if we had used AJCC staging.

Additional limitations to this study also include inability to control for other variables such as body mass index (BMI), genetic syndromes, and family history which may be associated with thyroid cancer risk [2]. Our use of Bonferroni correction to correct for multiple chi squared test in bivariate analysis may sometimes over correct, due to a resulting decrease in statistical power when employing Bonferroni corrections. Also, some of our results presented with wide confidence intervals which should be interpreted with caution. Finally, FCDS data, like most state-based surveillance systems has missing or incomplete data for occupation [18], leading to exclusion of a large proportion of the registry records (75.2%). Excluded tumor records were different from our study sample in age, gender, ethnicity, diagnostic year, tumor stage, and histologic type. This can result in over or underestimation of risks [48]. We present some notable differences in our analytic sample compared to excluded (missing occupation) sample especially for histologic type in Supplemental Table 1.

## Conclusion

Findings from this study suggest that the higher risk of thyroid cancer among firefighters is more likely due to differentiated thyroid cancer, more specifically papillary thyroid cancer compared to service, white-collar, blue-collar, and other occupational groups. There is no statistically significant difference in SEER-derived early versus late-stage diagnoses among firefighters relative to the other four occupational groups. Because differentiated thyroid cancers are more likely to have good prognosis and better treatment outcome, firefighters may benefit from routine annual screenings beyond their recommended employee baseline routine medical examination. Additionally, our findings prompt further investigations to examine the serum levels of EDCs and other chemical exposures of firefighters, in association with variants of thyroid cancer among firefighters.

## Supplementary Information

**Additional file 1: Supplemental table 1.** A comparison of excluded and included (analytic) records from the FCDS 1981-2014 for thyroid tumors.

**Additional file 2: Supplemental table 2. **Binary logistic regression showing the adjusted odds of histologic type, age, and stage at diagnosis of thyroid cancer among firefighters compared to other occupation groups in the FCDS 1981-2014; Thyroid tumor analytic dataset.

**Additional file 3: Supplemental table 3. **Adjusted multinomial logistic regression showing the odds of histologic types, age, and stage at diagnosis of thyroid cancer among white men in the FCDS 1981-2014; Thyroid tumor analytic dataset.

## Data Availability

Florida Cancer Data System linkage data are subject to privacy/ethical restrictions per registry policy; Access to underlying data would be subject to application to the FCDS for consideration.

## Abbreviations

AJCC: American Joint Committee on Cancer
FCDS: Florida Cancer Data System
FMO: Firefighter Marshals Office
NFPA: National Fire Protection Association
PBDE: Polybrominated diphenyl ether
SEER: Surveillance, Epidemiology, and End Results Program
T3: Triiodothyronine
T4: Thyroxine
TSH: Thyroid Stimulating Hormone
