# Supplementary material for: A comparative analysis of histologic types of thyroid cancer between career firefighters and other occupational groups in Florida
Source: BMC Endocr Disord. 2022 Sep 2;22:222. doi: 10.1186/s12902-022-01104-5 (PMC9438132; doi:10.1186/s12902-022-01104-5)
Supplement: Supplementary file 1 — Additional file 1: Supplemental table 1. A comparison of excluded and included (analytic) records from the FCDS 1981-2014 for thyroid tumors. [file 12902_2022_1104_MOESM1_ESM.docx]

| **Supplemental table 1: A comparison of excluded and included (analytic) records from the FCDS 1981-2014 for thyroid tumors** | | | |
| --- | --- | --- | --- |
| **Characteristics n (%)** | **Patients with thyroid cancer (n=40,672)** | | **p-value** ^d^ |
|  | **Included** | **Excluded ^a^** |  |
|  | n=8,291 (24.8%) | n=32,381 (75.2%) |  |
| **Age in years** **^b^** |  |  | <0.001 |
| Mean (SD, min, max) | 46.8 (12.4, 20, 69) | 54.7 (16.8, 20, 100) |  |
| **Age categories (years)** |  |  | <0.001 |
| Missing | - | 7,482 (23.1) |  |
| 18 to 29 | 828 (10.0) | 2,271 (7.0) |  |
| 30 to 49 | 3,804 (45.9) | 10,851 (33.5) |  |
| 50 to 69 | 3,659 (44.1) | 11,777 (36.4) |  |
| **Gender** |  |  | <0.001 |
| Male | 1,845 (22.3) | 8,964 (27.7) |  |
| Female | 6,446 (77.7) | 23,417 (72.3) |  |
| **Race** |  |  | 0.295 |
| White | 7,305 (88.1) | 28,545 (88.2) |  |
| Non-White | 938 (11.3) | 3,599 (11.1) |  |
| Unknown | 48 (0.6) | 237 (0.7) |  |
| **Ethnicity** |  |  | 0.018 |
| Hispanic | 1,543 (18.6) | 5,978 (18.5) |  |
| Non-Hispanic | 6,696 (80.8) | 26,094 (80.6) |  |
| Unknown | 52 (0.6) | 309 (1.0) |  |
| **Diagnosis year** |  |  | <0.001 |
| 1981- 1991 | 222 (2.7) | 4,512 (14.0) |  |
| 1992 - 2002 | 1,308 (15.8) | 8,561 (26.4) |  |
| 2003 - 2014 | 6,761 (81.6) | 19,308 (59.6) |  |
| **Tumor stage ^c^** |  |  | <0.001 |
| Early stage | 5,590 (67.4) | 20,664 (63.8) |  |
| Late stage | 2,331 (28.1) | 9,090 (28.1) |  |
| Unknown | 370 (4.5) | 2,627 (8.1) |  |
| **Histological type** |  |  | <0.001 |
| Papillary | 7,099 (85.6) | 24,849 (76.7) |  |
| Follicular | 476 (5.7) | 2,903 (9.0) |  |
| Rare type ^e^ | 716 (8.6) | 4,629 (14.3) |  |
| **^a^** Patients with missing values on either occupation or histologic subtype variables  **^b^** Age is age in years at cancer diagnosis; SD is standard deviation  **^c^** Based on SEER stage 2000, excludes stage 0, late stage includes regional and distant metastasis  ^d^ P-values are calculated either with Student’s t-test for continuous variables or with chi-squared test for independence and considered statistically significant when p-value <0.05  ^e^ Rare types include other less common/aggressive histologic types of thyroid cancer which includes oxyphilic (27%), medullary (21%), carcinoma NOS (20%), anaplastic (10%), other rare, and unknown | | | |
