# Supplementary material for: A comparative analysis of histologic types of thyroid cancer between career firefighters and other occupational groups in Florida
Source: BMC Endocr Disord. 2022 Sep 2;22:222. doi: 10.1186/s12902-022-01104-5 (PMC9438132; doi:10.1186/s12902-022-01104-5)
Supplement: Supplementary file 2 — Additional file 2: Supplemental table 2. Binary logistic regression showing the adjusted odds of histologic type, age, and stage at diagnosis of thyroid cancer among firefighters compared to other occupation groups in the FCDS 1981-2014; Thyroid tumor analytic dataset. [file 12902_2022_1104_MOESM2_ESM.docx]

| **Supplemental table 2: Binary logistic regression showing the adjusted odds of histologic type, age, and stage at diagnosis of thyroid cancer among firefighters compared to other occupation groups in the FCDS 1981-2014; Thyroid tumor analytic dataset** | | | |
| --- | --- | --- | --- |
| **Occupation groups ^c^** | **Histologic sub-types^1^** | | |
|  | **Differentiated ^b^  vs Rare types ^a^** | | |
|  | **aOR** | **95% CI** | **p-value** |
| Firefighters | 1.00 | - | - |
| Service | **0.19** | **0.06, 0.61** | **0.006** |
| White-collar | **0.23** | **0.07, 0.72** | **0.012** |
| Blue-collar | **0.18** | **0.05, 0.57** | **0.004** |
| Other | **0.23** | **0.07, 0.76** | **0.002** |
|  | **Age group (years) at diagnosis^2^** | | |
|  | **18 - 49 vs 50 - 69** | | |
| Firefighters | 1.00 | - | - |
| Service | **0.44** | **0.28, 0.67** | **<0.001** |
| White-collar | **0.38** | **0.25, 0.57** | **<0.001** |
| Blue-collar | **0.34** | **0.22, 0.52** | **<0.001** |
| Others | **0.41** | **0.27, 0.62** | **<0.001** |
|  | **Tumor stage^3^** | | |
|  | **Other ^d^ vs Late** | | |
| Firefighters | 1.00 | - | - |
| Service | 1.01 | 0.66, 1.52 | 0.983 |
| White-collar | 0.96 | 0.65, 0.84 | 0.839 |
| Blue-collar | 0.95 | 0.63, 1.44 | 0.814 |
| Others | 0.84 | 0.56, 1.26 | 0.396 |
| aOR: adjusted odds ratio; 95%CI: 95% confidence interval adjusted for ^1^ age, gender, race, tumor stage, and diagnosis year. ^2^ histologic type, gender, race, tumor stage, and diagnosis year ^3^ histologic type, age, gender, race, and diagnosis year  Significant p-value <0.05  **^a^** Rare types include other less common/aggressive histologic types of thyroid cancer which includes oxyphilic (27%), medullary (21%), carcinoma NOS (20%), anaplastic (10%), other rare, and unknown  **^b^** Differentiated histologic types include follicular, and papillary thyroid cancer  **^c^** Other occupation includes retired, students, housewife/homemakers, farm workers, and disabled  **^d^** Other stage at diagnosis include early and unknown stage | | | |
