# Supplementary material for: A comparative analysis of histologic types of thyroid cancer between career firefighters and other occupational groups in Florida
Source: BMC Endocr Disord. 2022 Sep 2;22:222. doi: 10.1186/s12902-022-01104-5 (PMC9438132; doi:10.1186/s12902-022-01104-5)
Supplement: Supplementary file 3 — Additional file 3: Supplemental table 3. Adjusted multinomial logistic regression showing the odds of histologic types, age, and stage at diagnosis of thyroid cancer among white men in the FCDS 1981-2014; Thyroid tumor analytic dataset. [file 12902_2022_1104_MOESM3_ESM.docx]

| **Supplemental table 3: Adjusted multinomial logistic regression showing the odds of histologic types, age, and stage at diagnosis of thyroid cancer among white men in the FCDS 1981-2014; Thyroid tumor analytic dataset** | | | | | | |
| --- | --- | --- | --- | --- | --- | --- |
| **Occupation groups ^a^** | **Histologic sub-types^1^** | | | | | |
|  | **Follicular vs Papillary** | | | **Rare ^b^ vs Papillary** | | |
|  | **aOR** | **95% CI** | **p-value** | **aOR** | **95% CI** | **p-value** |
| Firefighters | 1.00 | - | - | 1.00 | - | - |
| Service | 0.35 | 0.11, 1.12 | 0.075 | **3.73** | **1.05, 13.32** | **0.043** |
| White-collar | 0.80 | 0.36, 1.76 | 0.578 | 2.93 | 0.89, 9.65 | 0.077 |
| Blue-collar | 0.92 | 0.40, 2.13 | 0.854 | 3.33 | 0.99, 11.21 | 0.052 |
| Other | 0.56 | 0.18, 1.74 | 0.320 | 3.35 | 0.91, 12.32 | 0.069 |
|  | **Age group (years) at diagnosis^2^** | | | | | |
|  | **18 - 29 vs 50 - 69** | | | **30 - 49 vs 50 - 69** | | |
| Firefighters | 1.00 | - | - | 1.00 | - | - |
| Service | 0.30 | 0.10, 0.92 | 0.035 | 0.61 | 0.34, 1.06 | 0.081 |
| White-collar | **0.30** | **0.13, 0.69** | **0.004** | **0.47** | **0.29, 0.75** | **0.002** |
| Blue-collar | **0.20** | **0.08, 0.52** | **0.001** | **0.39** | **0.24, 0.65** | **0.000** |
| Other | 2.14 | 0.89, 5.13 | 0.089 | **0.37** | **0.20, 0.69** | **0.002** |
|  | **Tumor stage ^3^** | | | | | |
|  | **Early vs Late** | | | **Unknown vs Late** | | |
| Firefighters | 1.00 | - | - | 1.00 | - | - |
| Service | 1.21 | 0.69, 2.11 | 0.505 | 7.66 | 0.80, 73.37 | 0.077 |
| White-collar | 0.97 | 0.61, 1.53 | 0.886 | 5.40 | 0.65, 44.64 | 0.118 |
| Blue-collar | 0.85 | 0.53, 1.38 | 0.516 | 4.96 | 0.58, 42.29 | 0.143 |
| Other | 1.14 | 0.64, 2.05 | 0.656 | **9.65** | **1.07, 86.76** | **0.043** |
| aOR: adjusted odds ratio; 95%CI: 95% confidence interval adjusted for ^1^ age, gender, race, tumor stage, and diagnosis year. ^2^ histologic type, gender, race, tumor stage, and diagnosis year ^3^ histologic type, age, gender, race, and diagnosis year  Significant p-value <0.05  **^a^** Other occupation includes retired, students, housewife/homemakers, farmers, and disabled  **^b^** Rare types include other less common/aggressive histologic types of thyroid cancer which includes oxyphilic (27%), medullary (21%), carcinoma NOS (20%), anaplastic (10%), other rare, and unknown  Wide confidence intervals is as a result of small sample size and should be interpreted with caution | | | | | | |
